# Polymicrobial synergy within oral biofilm promotes invasion of dendritic cells and survival of consortia members

**DOI:** 10.1038/s41522-019-0084-7

**Published:** 2019-03-18

**Authors:** Ahmed El-Awady, Mariana de Sousa Rabelo, Mohamed M. Meghil, Mythilypriya Rajendran, Mahmoud Elashiry, Amanda Finger Stadler, Adriana Moura Foz, Cristiano Susin, Giuseppe Alexandre Romito, Roger M. Arce, Christopher W. Cutler

**Affiliations:** 1 Department of Periodontics, The Dental College of Georgia, Augusta University, Augusta, GA USA; 2 Immunology Program, Department of Research, Cancer Childrens Hospital, Cairo, 57357 Egypt USA; 3 Department of Stomatology, Division of Periodontics, School of Dentistry, University of São Paulo, São Paulo, Brazil; 4 Department of Oral Biology, The Dental College of Georgia, Augusta University, 30912 Augusta, Georgia

**Keywords:** Cellular microbiology, Biofilms, Cellular microbiology, Biofilms

## Abstract

Years of human microbiome research have confirmed that microbes rarely live or function alone, favoring diverse communities. Yet most experimental host-pathogen studies employ single species models of infection. Here, the influence of three-species oral microbial consortium on growth, virulence, invasion and persistence in dendritic cells (DCs) was examined experimentally in human monocyte-derived dendritic cells (DCs) and in patients with periodontitis (PD). Cooperative biofilm formation by *Streptococcus gordonii*, *Fusobacterium nucleatum* and *Porphyromonas gingivalis* was documented in vitro using growth models and scanning electron microscopy. Analysis of growth rates by species-specific 16s rRNA probes revealed distinct, early advantages to consortium growth for *S. gordonii* and *F. nucleatum* with *P. gingivalis*, while *P. gingivalis* upregulated its short mfa1 fimbriae, leading to increased invasion of DCs. *F. nucleatum* was only taken up by DCs when in consortium with *P. gingivalis*. Mature consortium regressed DC maturation upon uptake, as determined by flow cytometry. Analysis of dental plaques of PD and healthy subjects by 16s rRNA confirmed oral colonization with consortium members, but DC hematogenous spread was limited to *P. gingivalis* and *F. nucleatum*. Expression of *P. gingivalis* mfa1 fimbriae was increased in dental plaques and hematogenous DCs of PD patients. *P. gingivalis* in the consortium correlated with an adverse clinical response in the gingiva of PD subjects. In conclusion, we have identified polymicrobial synergy in a three-species oral consortium that may have negative consequences for the host, including microbial dissemination and adverse peripheral inflammatory responses.

## Introduction

The human microbiome project has revealed the enormous diversity of the oral microbiome.^1,2^ Molecular and microbiological studies reported more than 700 species in the oral cavity with more than 500 within the subgingival biofilm.^3,4^ This diversity has been recognized through decades of microbiological studies;^5–10^ however, much still needs to be done to understand inter-species interactions, through synergy or antagonism, and the role of this interaction in inflammatory dysbiosis.^11^ Early studies revealed significant differences in the microbes that colonize the oral mucosa in healthy and periodontitis (PD) patients.^12,13^ Cross sectional and association studies identified putative pathogens in PD. Most notable are *P. gingivalis*, *Treponema denticola* and *Tannerella forsythia*, based on their virulence factors and strong association with diseased sites.^9,14^ The early interpretation of these findings was that the transition from health to destructive periodontitis is caused by emergence of specific pathogens within oral biofilm. More recent evidence implicates ecological disruption of the commensal oral biofilm in the pathological transition, i.e., dysbiosis.^15–18^ Although the evidence of microbiome impact on the human health and disease conditions is well established, presently unclear is how the oral microbiome influences local and systemic health. *P. gingivalis* has been termed a keystone pathogen due to its ability to orchestrate a dysbiosis,^21^ presumably through expression of specific virulence factors^19–21^ that modulate innate immunity.^22,23^ For example, in macrophages, *P. gingivalis* subverts nitric oxide synthase (iNOS)-dependent killing in vitro and in vivo by stimulation of cAMP production.^24^ In dendritic cells (DCs), *P. gingivalis* employs adhesive fimbriae to manipulate TLR activation, as well as activate cross talk of TLR2 with C-type lectin receptor DC-SIGN on.^17,25,26^ This enables *P. gingivalis* to evade innate immune recognition and sustain disease progression in a susceptible host. Indeed, *P. gingivalis* can initiate bone destruction upon inoculation into the oral cavity of animals such as mice and nonhuman primates;^27,28^ however, recent studies in a germ-free mouse suggest that the existing flora may be required for *P. gingivalis* pathogenesis.^29^ Consistent with this notion, very low inoculum (less than 0.01%) with *P. gingivalis* induces a dysbiosis and promotes alveolar bone loss in mice.^29^

*P. gingivalis* has been shown to invade myeloid DCs with its glycoprotein Mfa1 fimbriae, routing it into non-autophagosomal compartments.^30^ This routing enables *P. gingivalis* to evade the intracellular degradation by autophagy machinery and increase its survival within DCs.^30^ Due to early indications that *P. gingivalis* fimbrial expression is under environmental control,^31^ combined with the role of mfa1 fimbriae invasion of DCs in vivo^45^ and survival inside DCs in vitro,^30^ we hypothesized that polymicrobial conditions would influence the mfa1 expression. We developed a polymicrobial consortium model, involving growth of *P. gingivalis* with commensal early colonizer *Streptococcus gordonii (S. gordonii)*, and *Fusobacterium nucleatum (F. nucleatum)*. In addition, those three species were analyzed in plaque and circulating blood cells in chronic periodontitis patients. Therefore, the influence of this three-species consortium on biofilm colonization and on DC invasion in vitro and in vivo was investigated. Our results indicate that growth of *P. gingivalis* with *S. gordonii*, and *F. nucleatum* facilitates biofilm formation and increases expression of *mfa-1*; which subsequently promotes invasion of DCs by *P. gingivalis* and *F. nucleatum* and impairs DC maturation. Analysis of subgingival biofilm samples (i.e., plaque) from PD patients vs. healthy controls indicates an increase in *P. gingivalis* and its encoded mfa-1 fimbriae in PD plaques. Circulating DCs in PD patients showed increased carriage of *F. nucleatum* and *P. gingivalis*, with higher expression of *mfa-1*. Strikingly, the majority of microbial carriage in these patients was contained within blood DCs compared to the remaining PBMCs. Overall our results support an important function for polymicrobial synergy in invasion and survival of consortia members in dendritic cells in vitro and in vivo, leading to microbial dissemination and disruption of immune homeostasis.

## Results

### Consortium growth promotes biofilm formation and proliferation of each species

**Fig. 1:**
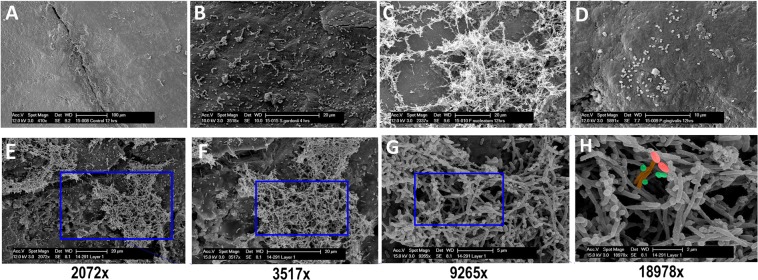
Colonization and growth advantages in consortium in-vitro. SEM scans showing biofilm formation after 12 h cultured on sterile tooth surface: **a** Control (No bacteria), **b** *S. gordonii* alone, **c** *F. nucleatum* alone, **d** *P. gingivalis*, and **e**–**h** Three species mixed biofilm (Lower magnification) to (Higher magnification) showing the characteristics rod-shaped *P. gingivalis*, cocci *S. gordonii* and fusiform rods *F. nucleatum* forming aggregate structure. **e**–**i** show different magnification of the same section with right at the lowest and left with at higher magnification. Blue boxes mark the sequenced higher magnifications from (**e**–**h**). **h** Computer-generated image shows color markings for rod-shaped *P. gingivalis* as red, cocci *S. gordonii* as green and fusiform rods *F. nucleatum* as orange (photoshop CS6). The three species frequently embedded within a self-produced matrix (**e** and **f**) Individual magnifications and size bars are shown on labels of each micrograph

Initial growth curves were established for log growth phase of *S. gordonii*, *F. nucleatum* and *P. gingivalis* in broth cultures. The ability of these three strains to form biofilm was tested, alone and in consortium, on sterile extracted human teeth (Fig. 1). Bacteria were grown on teeth surfaces for 12 h and biofilm were evaluated using scanning electron microscopy (SEM). SEM images show control tooth surface with no bacteria (Fig. 1a) and *S. gordonii* alone formed a coating or biofilm on the root surface (Fig. 1b). *F. nucleatum* alone formed a loose filamentous network (Fig. 1c), while *P. gingivalis* was sparse on root surfaces after 12 h of incubation (Fig. 1d). Early colonizer *S. gordonii* was transferred to sterile extracted human teeth, and after 2 h, bridge species *F. nucleatum* was added, followed by pathobiont *P. gingivalis* in 1 aerobic (*S. gordonii)*: 10 anaerobic: (*F. nucleatum* + *P. gingivalis*). The three species together formed a well-organized biofilm structure or consortium (Fig. 1e–h), similar to that previously observed in *ex vivo* isolated plaque samples collected from root surfaces at chronic periodontitis sites.^5^ These bacterial aggregates contain all three morphological features of *P. gingivalis* as rod-shaped, *S. gordonii* as cocci and *F. nucleatum* as fusiform rods (Fig. 1e–h).

**Fig. 2:**
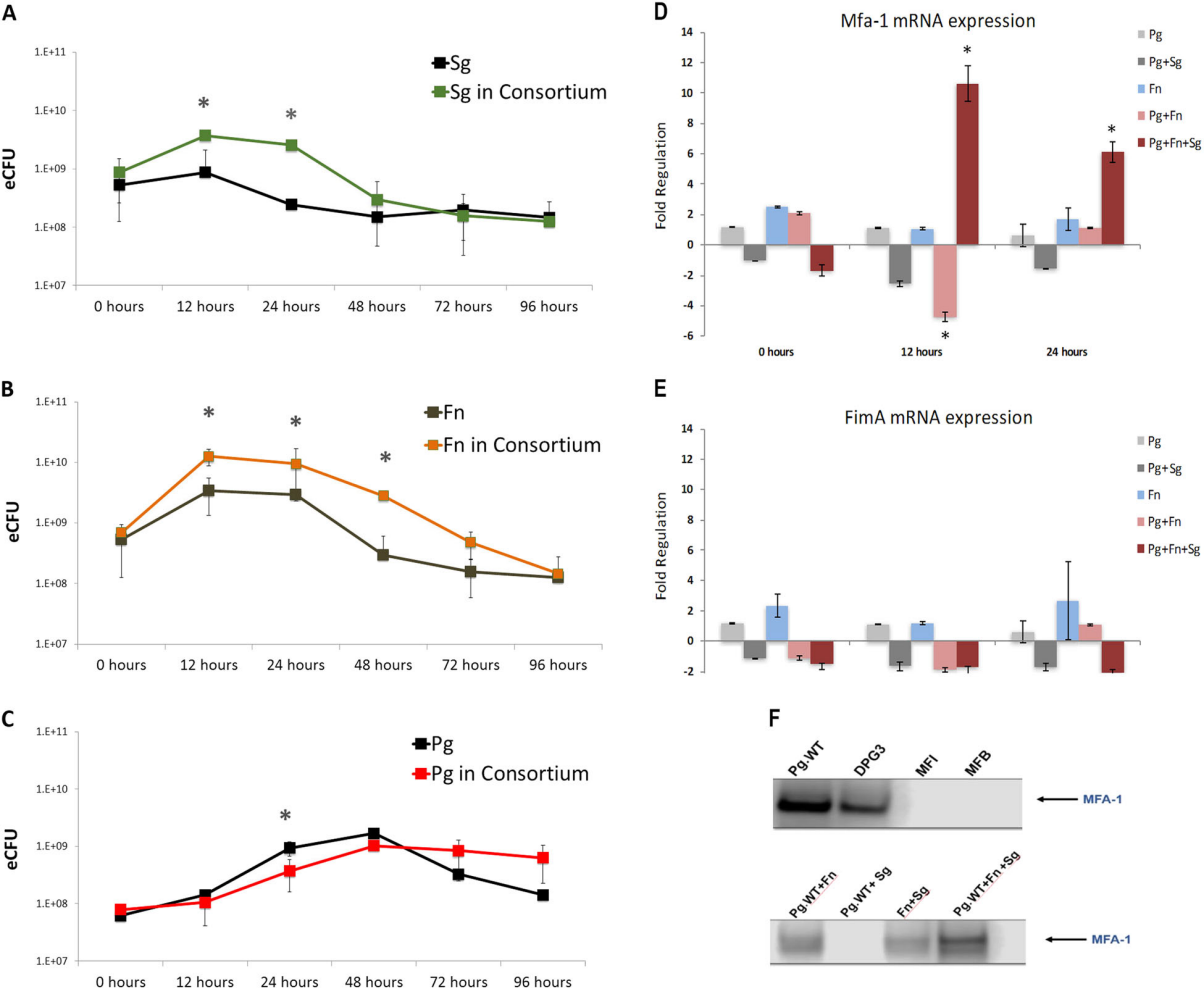
The effect of consortia on growth behavior of each species and fimbriae expression of Pg. Estimated colony forming (eCFU) units of each species grown alone or in consortium from 0–96 h: **a** Growth curve for *S. gordonii* (Sg) alone (black) and within consortium (Red). **b** *F. nucleatum* (Fn) alone (Black) and within consortium (Orange). **c** *P. gingivalis* (Pg) alone (blue) and within consortium (Red). For eCFU calculation, standard curves were generated for cycle threshold versus CFU values and regression analysis were carried out for each serial dilution to estimate CFU based on 16s rRNA expressions (detailed in the methods section). Fold regulation of mRNA expression of *mfa-1* **d** and *fimA* **e** of *P. gingivalis* (Pg) grown alone, in incomplete two-species or complete three-species consortium. Fold regulations were quantified relative to controls (*P. gingivalis* alone) using (2^−ΔΔCT^) method and 16s rRNA was used as housekeeping gene. Statistically significant increase in the expression of *mfa1* was detected after 12 and 24 h (mature complete consortium). No significant change was detected in the expression of *mfa1* in incomplete consortium (two species) except downregulation when *P. gingivalis* was co-cultured with *F. nucleatum* **d**. The expression of *fimA* was stable during 24 h with no significant change in incomplete and complete consortium **e**. Standard laboratory and isogenic fimbriae-deficient mutant strains of *P. gingivalis*, including: WT Pg381 (Mfa1 + FimA+), DPG3 (Mfa1 + FimA−), MFI (Mfa1−FimA+) and MFB (Mfa1-FimA−) were probed for Mfa1 protein expression by Western blot using monoclonal antibody to Mfa-1 **f** (upper panel) corroborating specificity of antibody. Mfa1 protein expression by WT Pg381 grown with Fn (lane 1), Sg (lane 2), Sg + Fn (lane 3) and complete consortium PgWT + Fn + Sg for 12 h showing differential regulation of Mfa1

To determine how consortium growth in planktonic culture affected proliferation of each species, a regression analysis, relating CFU to PCR threshold cycles (Ct) for each species was developed. This facilitated an estimation of CFU (eCFU) for each species grown alone or in complete consortia (detailed in the method section). *S. gordonii* and *F. nucleatum* demonstrated significant increases in eCFU in consortium. This significant shift was detected at 12 and 24 h for *S. gordonii* and at 12, 24 and 48 h for *F. nucleatum* (Fig. 2a, b). On the other hand, consortium growth did not benefit *P. gingivalis* for up to 48 h, although consortium growth trended towards favoring increased *P. gingivalis* eCFU at 72 and 96 h (Fig. 2c).

#### Complete consortium promotes Increased *mfa-1* expression on *P. gingivalis* in vitro

As *P. gingivalis* fimbriae play critical roles in colonization and binding to other biofilm species (reviewed in^11^) we analyzed the level of expression of both the (minor) *mfa-1* and (major) *fimA* fimbriae by *P. gingivalis* grown alone, in two-species (incomplete) and three-species (complete) consortia in vitro. *mfa-1* mRNA expression was quantified by qrt-PCR during consortium maturation, from 0 to 12 to 24 h. Significant increases in *mfa-1* were detected at 12 and 24 h in complete consortium (Fig. 2d and Supplemental Table 1), consistent with decreased DC maturation (Figs. 4f, m). No significant changes in *mfa-1* expression, at the mRNA level, were detected when *P. gingivalis* was grown in incomplete consortia with Pg-Sg, although there was a downregulation of *mfa1* at 12 h of Pg-Fn consortium (Fig. 2d). Major fimbriae (*fimA*) expression was maintained during biofilm maturation with no significant changes (Fig. 2e). Western blot using our monoclonal antibody to native Mfa1 (confirmed high expression of Mfa-1 protein at 12 h of maturation in the complete consortium *Pg*-*Sg-Fn*, but not incomplete consortium *Pg-Fn* or *Pg-Sg* (Fig. 2f). Indeed, our Western blotting results suggest that this Sg strain under these conditions, negatively regulates Mfa1 expression. While the mechanism needs to be established, it is consistent with Sg mediated regulation of the *mfa1* gene by FimR directly binding to the promoter region of *mfa1*.^32^ Unexpectedly, we also observed cross-reactivity of Fn and Sg with the AEZaMfa1 mAB, previously generated by our laboratory to the native Mfa1 glycosylated protein,^33^ possibly due to immunoreactivity with fucose, mannose, N-acetylglucosamine or N-acetylgalactosamine residues,^34^ which may be present on Fn and Sg.

### Consortium growth promotes uptake of consortium members and intracellular persistence

**Fig. 3:**
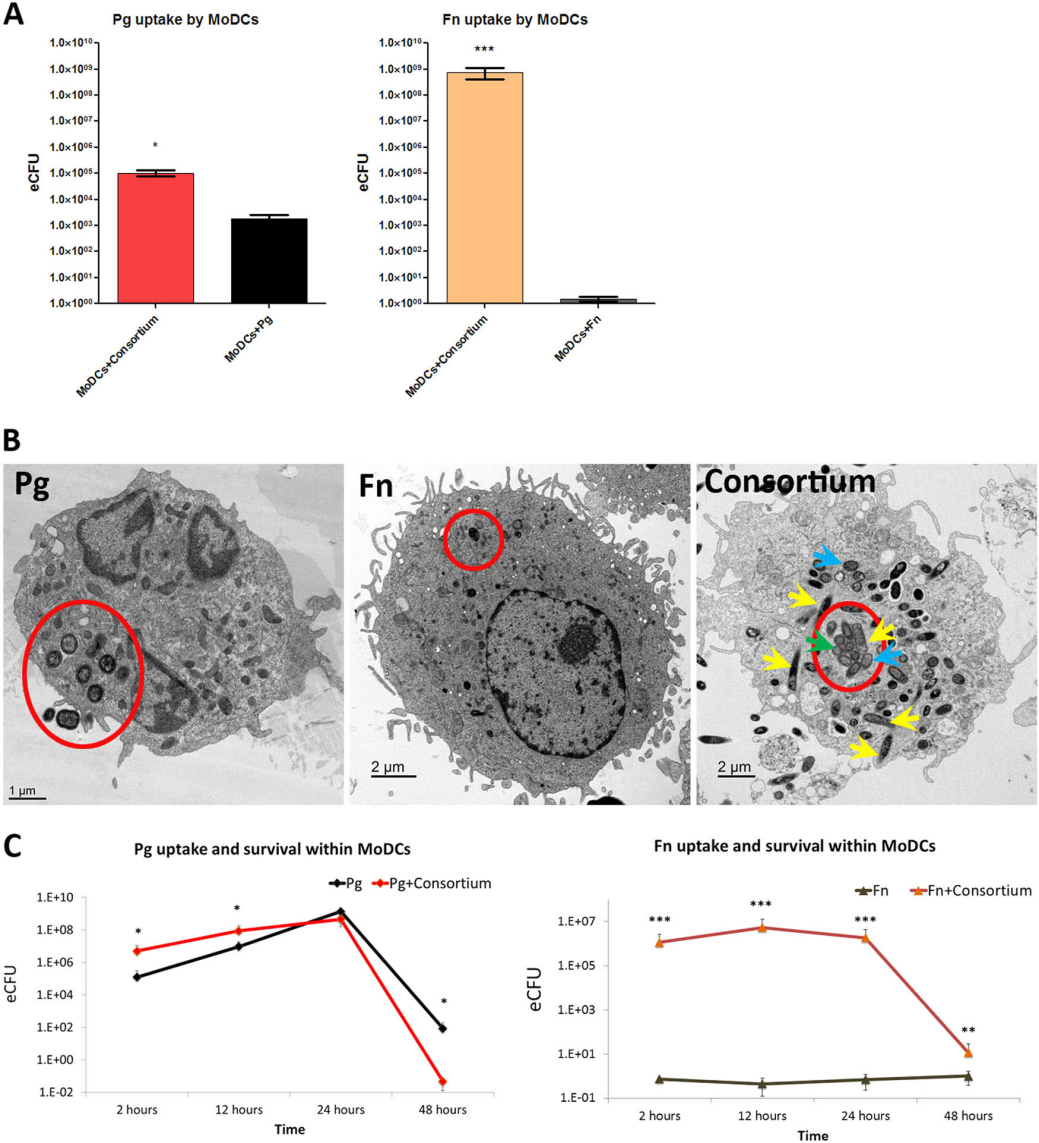
Complete consortium influences uptake and survival of *P. gingivalis* and *F. nucleatum* in MoDCs. **a** Shows uptake in eCFU of Pg (*P. gingivalis)* (Left) and Fn (*F. nucleatum)* (Right) grown in the presence or absence of consortium in MoDCs). Statistically significant higher eCFU of Pg was detected within MoDCs when infected with consortium for 12 h (eCFU of Pg alone = 1.79E + 03 ± 1.45E + 03) (eCFU of Pg with consortium = 1.04E + 05 ± 4.84E + 04). In addition, minimal to no detected eCFU within MoDCs when infected by Fn alone (eCFU = 1.50E + 00 ± 5.00E-01). Substantial increase in eCFU of Fn within MoDCs infected by consortium for 12 h (eCFU = 7.51E + 08 ± 6.12E + 08). **b** TEM images showing MoDCs uptake of *P. gingivalis* (rod-shaped) alone (Left), no uptake of *F. nucleatum* (middle), and uptake of *P. gingivalis* (rod-shaped) and *F. nucleatum* (long rods) and *S. gordonii* (several cocci) (Right) grown in complete consortium. **c** Uptake and survival of *P. gingivalis* (Left) and *F. nucleatum* (Right) grown alone or in consortium, by eCFU at 2, 12, 24 and 48 h within MoDCs. * Statistically significant difference with *P* < 0.05 and *** Statistically significant difference with *P* < 0.001)

As Mfa-1 is required for efficient uptake of *P. gingivalis* by DCs, and is upregulated in the complete consortium, we hypothesized that consortium growth would increase uptake of *P. gingivalis* by MoDCs. Indeed, when grown in consortium, *P. gingivalis* was more efficiently taken up by MoDCs with eCFU of (1.04E + 05 ± 4.84E + 04), relative to *P. gingivalis* alone at 12 h (1.79E + 03 ± 1.45E + 03) (Fig. 3a). Moreover, *F. nucleatum* was not taken up by MoDCs, unless in a consortium (eCFU; 7.51E + 08 ± 6.12E + 08) within MoDCs (Fig. 3a). This was confirmed by transmission electron microscopy (TEM) showing intracellular *F. nucleatum* only when in consortium at 12 h, as determined by morphology (>2 μm long rods, yellow arrows) (Fig. 3b). Shown is a cluster consisting of several cocci (0.5 μm *S. gordonii*, *green arrow*), short rod (0.5 x 1 μm *P. gingivalis*, blue arrow) and Fn (Fig. 3b). Persistence of *P. gingivalis* and *F. nucleatum* from 0–24 h were assessed in lysed MoDCs at these time points. The results confirm more efficient uptake and persistence of *P. gingivalis* in a consortium by MoDCs at up to 12 h, followed by a decline. In contrast, *F. nucleatum* was only taken up in a consortium and persisted for up to 24 h, followed by a decline (Fig. 3c).

### Time in consortium growth impedes MoDC maturation

**Fig. 4:**
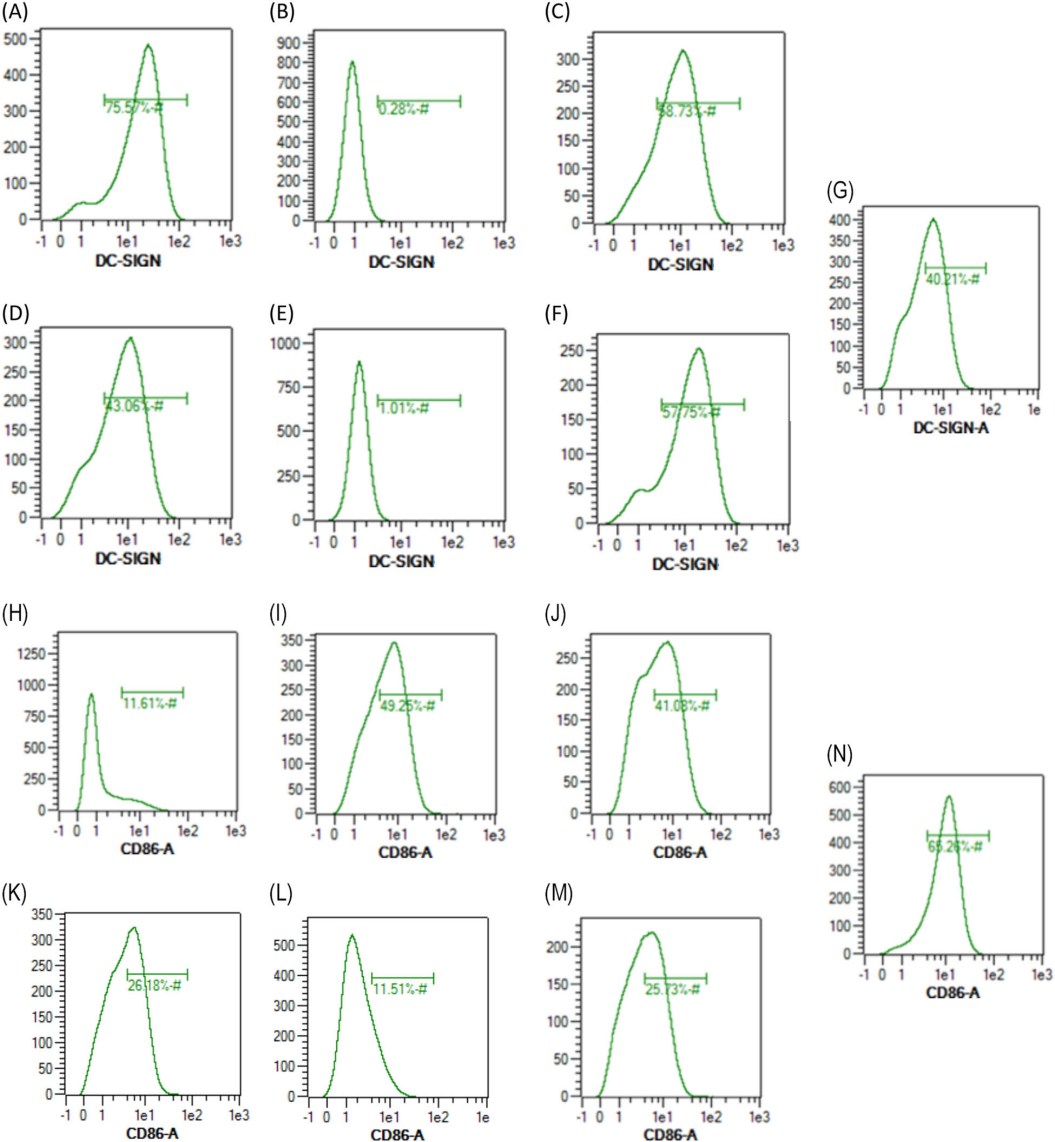
Age of consortium influences DC maturation markers. Representative histograms from flow cytometric analysis of %DC-SIGN (**a–g**) and %CD86 (**h**–**n**) expression by CD1C + MoDCs after co-culture at 10:1 MOI for 12 h with: **a**, **h** no bacteria (control), **b**, **i** WTPg381, **c**, **j** Fn, **d**, **k** Sg, **e**, **l** immature (day 0), **f**, **m** mature (24 h) consortium and **g**, **n** maturation inducer E.coli LPS/TNFα, as reported^63^

*P. gingivalis* has previously been reported to inhibit or activate DC maturation, depending on whether Mfa1 or FimA, respectively, was expressed by the isogenic fimbriae mutant Pg strain used.^30,35^ Here we looked at whether time in consortium growth would influence immaturity marker DC-SIGN and maturity/costimulatory marker CD86. Control immature MoDCs were 75.57% positive for Pg uptake mediator and immaturity marker DC-SIGN^30^ (Fig. 4a) and 11.61% positive for maturity/costimulatory marker CD86, (Fig. 4h). WT Pg381 alone at 10 MOI was a potent down regulator of DC-SIGN (0.28% DC-SIGN+) consistent with internalization of DC-SIGN,^34^ but was not a potent upregulator of CD86, relative to LPS/TNFa (Fig. 4n), suggesting a semi-mature DC status. Mature consortium retained MoDCs in a more immature status (57.5% DC-SIGN+, 25.73% CD86+) (Fig. 4f, m), consistent with mfa1 expression levels (Fig. 2d, f). The interactions of MoDCs with consortia members was captured by time lapse video, wherein MoDCs were infected with immature (Supplementary video 1) and mature consortium (Supplementary video 2). In Supplementary video 1, human MoDCs were observed actively capturing bacteria within the first 2–3 h of co-culture, then showed morphological characteristics of maturing DCs, formation of dendrites. Furthermore, late in the 24 h incubation, MoDCs start to form cell-to-cell conjugates, not unlike those described previously in periodontal tissues^36^ (Supplementary video 1). In Supplementary video 2, matured consortium (microbes co-cultured for 12 h) resulted in less active MoDCs with minimum percentage of cells actively engaging microbes. Neither cell-to-cell nor co-aggregation could be detected for the recorded 24 h (Supplementary video 2).

### Consortia in oral biofilms and in circulating blood DCs of PD patients

**Fig. 5:**
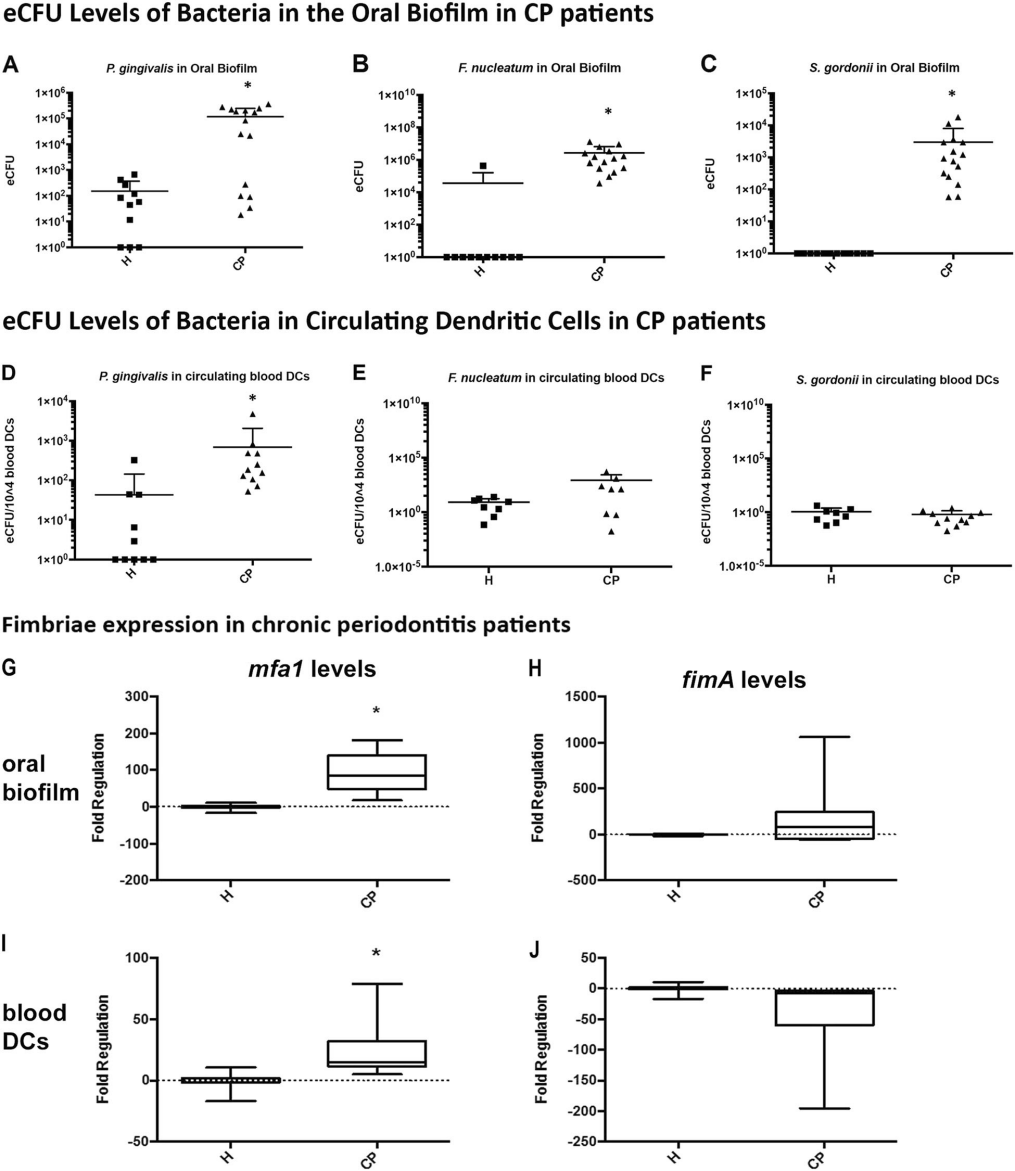
Detection of consortia in oral biofilms and in circulating blood DCs of PD patients. Plaque samples from healthy or PD patients were analyzed for eCFU levels of *P. gingivalis* **a**, *F. nucleatum* **b** and *S. gordonii* **c**. Peripheral blood DCs from healthy or PD patients were analyzed for eCFU levels of *P. gingivalis* **d**, *F. nucleatum* **e** and *S. gordonii* **f**. Oral plaque and blood samples were collected from 15 healthy individuals and 15 PD patients (2 healthy samples were excluded due to low RNA quality and quantity). Estimated colony forming units (eCFU) for each strain (*P. gingivalis*, *F. nucleatum* and *S. gordonii)* were calculated using regression analysis (detailed in material and method section). *Statistically significant difference at *P* < 0.05. **g** mRNA expression of *mfa-1* and *fimA* **h** in oral biofilm samples collected from healthy and PD gingival sulcus and in circulating blood DCs **i** and **j**. Fold regulations were quantified relative to controls (Healthy samples) using (2^-ΔΔCT^) method and 16s rRNA was used as housekeeping gene. Oral plaque and blood samples were collected from 15 healthy individuals and 15 PD patients (2 healthy samples were excluded due to low RNA quality and quantity). Gene expression plotted as fold regulation of PD samples relative to healthy controls. * Statistically significant difference at *P* < 0.05

Species specific 16s rRNA primers for each bacterial species were used to quantitate consortia members in subgingival oral biofilms (plaque) and in blood DCs isolated from PD patients. Our previous studies have identified blood DCs as hematogenous carriers of microbes in PD.^33^ Here we detected *F. nucleatum, P. gingivalis* and *S. gordonii* eCFU in oral biofilm samples as well as in circulating DCs of PD patients (Fig. 5) (Table 1). Statistically significant increases in eCFU of *P. gingivalis*, *F. nucleatum* and *S. gordonii* were detected in oral biofilm of PD patients. However, in circulating DCs of PD patients vs healthy controls only *P. gingivalis* was increased (Fig. 5) (Table 2). Although the carriage of *F. nucleatum* within circulating DCs was higher it did not achieve the statistical significance. Interestingly, eCFU of *S. gordonii* increased only in the oral biofilm with no change in the circulating DCs of PD patients (Fig. 5) (Tables 1 and 2). Next, we analyzed the correlation of eCFU to the clinical severity of PD in these patients. Correlation analyses of eCFU in oral biofilm and periodontal indices (probing pocket depth and bleeding on probing) were calculated. Only *P. gingivalis* eCFU significantly correlated to worsening of periodontal indices probing depths and bleeding on probing (Tables 3 and 4).

**Table 1:** Comparison between consortium eCFU in oral biofilm of PD patients

	Pg		Fn		Sg	
	H	PD	H	PD	H	PD
Avg	8.18E + 01	2.60E + 06	1.97E + 00	1.83E + 06	6.77E-04	1.48E + 04
Stdv	±4.6E + 01	±8.0E + 05	±6.6E-01	±4.5E + 05	±2.5E-04	±6.5E + 03
95% confidence interval	−4326000 to −876800		−2802000 to −849500		−28770 to −858.5	
*P* value	*0.0060		*0.0013		*0.0390	

Comparison of eCFU of consortium between healthy (H) group and chronic periodontitis (PD) patients was carried out using unpaired *t* test with Welch’s correction. Plaque samples were collected form 15 healthy and 15 chronic periodontitis patients, 2 healthy samples were excluded from the analysis due to low RNA quality and quantity

*H* healthy, *PD* chronic periodontitis, *eCFU* estimate colony forming unit, *Pg* *P. gingivalis*, *Sg* *S. gordonii*, *Fn* *F. nucleatum*, *Avg* average, *Stdv* standard deviation

**Table 2:** Comparison between consortium eCFU in circulating blood dendritic cells of CP patients

	Pg		Fn		Sg	
	H	PD	H	PD	H	PD
Avg	4.28E + 01	1.35E + 03	7.08E + 00	5.84E + 02	1.07E + 00	6.34E-01
Stdv	±3.1E + 01	±5.5E + 02	±2.7E + 00	±4.3E + 02	±3.7E-01	±2.0E-01
95% confidence interval	−2540 to −82.87		−1536 to 381.7		−0.4712 to 1.345	
*P* value	*0.0385		0.2121		0.3197	

Comparison of eCFU of consortium between healthy (H) group and chronic periodontitis (PD) patients was carried out using unpaired *t* test with Welch’s correction. Plaque samples were collected form 15 healthy and 15 chronic periodontitis patients, 2 healthy samples were excluded from the analysis due to low RNA quality and quantity

*H* healthy, *PD* chronic periodontitis, *eCFU* estimate colony forming unit, *Pg* P. gingivalis, *Sg* S. gordonii, *Fn* F. nucleatum, *Avg* average, *Stdv* standard deviation

**Table 3:** Correlation between eCFU of *P. gingivalis* in oral biofilm and bleeding on probing

Correlations				
			Oral biofilm Pg	BOP (%)
Spearman’s rho	Oral biofilm Pg	Correlation coefficient	1000	.485^*^
		Sig. (2-tailed)	–	.026*
		*N*	21	21
	BOP (%)	Correlation coefficient	.485^*^	1000
		Sig. (2-tailed)	.026	–
		*N*	21	21

*eCFU* estimate colony forming unit, *Pg* *P. gingivalis*, *BOP* bleeding on probing

*Correlation is significant at the 0.05 level (2-tailed)

**Table 4:** Correlation between eCFU of *P. gingivalis* in oral biofilm and probing pocket depth

Correlations				
			Oral biofilm Pg	PPD
Spearman’s rho	Oral biofilm Pg	Correlation coefficient	1000	.618**
		Sig. (2-tailed)	–	.003
		*N*	21	21
	PPD	Correlation coefficient	.618**	1000
		Sig. (2-tailed)	.003	
		*N*	21	21

*eCFU* estimate colony forming unit, *Pg* *P. gingivalis*, *PPD* pocket depth

*Correlation is significant at the 0.05 level (2-tailed)

To determine whether oral carriage translated to hematogenous spread, correlation of eCFU in oral and blood DCs was determined. Significant correlations were only detected between eCFU of *P. gingivalis* in oral biofilms and blood DCs (Table 5). *P. gingivalis* eCFU in oral biofilm did not affect the circulating *F. nucleatum* nor *S. gordonii* eCFU. Oral *F. nucleatum* and *S. gordonii* eCFU did not show significant correlation to circulating microbiome (Table 5).

**Table 5:** Correlation between eCFU of *P. gingivalis* (Pg) in oral biofilm and circulating blood DCs in PD patients

Correlations				
			Oral biofilm Pg	DCs-Pg
Spearman’s rho	Oral biofilm Pg	Correlation coefficient	1000	.641**
		Sig. (2-tailed)	–	.002
		*N*	21	21
	Circulating DCs Pg	Correlation coefficient	.641**	1000
		Sig. (2-tailed)	.002	.
		*N*	21	21

*eCFU* estimate colony forming unit, *Pg* *P. gingivalis*, *DCs* dendritic cells

**Correlation is significant at the 0.01 level (2-tailed)

#### Influence of oral biofilm and circulating blood DC microenvironment on fimbriae expression

Previously, we reported that the *P. gingivalis* minor fimbriae (Mfa-1) was necessary for invasion and survival within human myeloid DCs.^30^ In the current study, we detected significant increases in the expression of mfa-1 mRNA within complete consortium in vitro. Hence, we quantified *mfa-1* and *fimA* mRNA expression in oral biofilm (Fig. 5g, h) and circulating DCs (Fig. 5i, j) of PD patients relative to healthy controls. Significant increase of *mfa-1* mRNA was detected in the oral biofilm of PD patient (Fig. 5g). In addition, the *mfa-1* carriage within DCs was significantly higher in PD (Fig. 5h). Among PD patients, the fold increase of *mfa-1* in the oral biofilm was significantly associated with its level in circulating DCs (Table 6). In contrast, expression of *fimA*, did not change significantly in the oral biofilm nor in circulating DCs of PD patients (Fig. 5i, j).

**Table 6:** Correlation between fold increase of Mfa-1 within the oral biofilm and within circulating blood DCs in PD patients

Correlations				
			mfa-1 (FR)	mfa-1 (FR)-DCs
Spearman’s rho	mfa-1 (FR)	Correlation coefficient	1000	.811**
		Sig. (2-tailed)	–	.001
		*N*	12	12
	mfa-1 (FR)-DCs	Correlation coefficient	.811**	1000
		Sig. (2-tailed)	.001	–
		*N*	12	12

*FR* fold regulation, *DCs* dendritic cells

**Correlation is significant at the 0.01 level (2-tailed)

### Blood myeloid DCs carry the majority of circulating consortia in PD patients

**Fig. 6:**
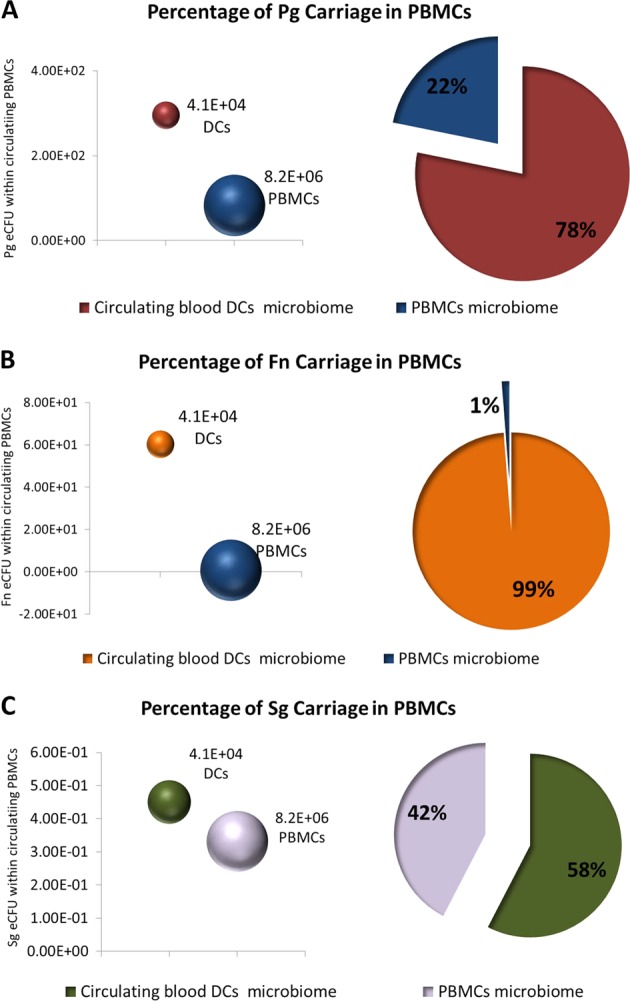
Estimation of the relative carriage rate of oral consortia in circulating blood DCs and PBMCs. **a** Pie-chart shows the percentage of eCFU of *P. gingivalis* carried by circulating DCs (deep red) and by PBMCs cells (dark blue) in chronic periodontitis patients. The percentages were calculated based on the ratio of eCFU within DCs and the total eCFU within both cells (DCs and PBMCs). Bubble graph shows eCFU of *P. gingivalis* within circulating DCs (deep red bubble) and within PBMCs cells (dark blue bubble) in PD patients considering the number of DCs and PBMCs represented by the bubble size. In the bubble chart, Y axis shows eCFU of *P. gingivalis* within DCs and PBMCs. Despite larger number of PBMCs (8.2 × 10^6) but they carry lower eCFU (8.24 × 10^1, blotted in Y axis) while low circulating DCs number (4.16 × 10^4) carries higher and the majority of eCFU of *P. gingivalis* (2.95 × 10^2, blotted in Y axis). **b** and **c** same calculation and blot were used to represent the carriage of *F. nucleatum* and *S. gordonii* within DCs and PBMCs in chronic periodontitis patients. PBMCs (8.2 × 10^6) carry eCFU (7.5 × 10^-01) of *F. nucleatum* (dark blue bubble, **b**) while circulating DCs (4.16 × 10^4) carry eCFU (6.0 × 10^01) of *F. nucleatum* (orange bubble, **b**). In another word, circulating DCs (4.16 × 10^4) carry 99% of *F. nucleatum* carriage within total circulating PBMCs (B, bi chart). **c** PBMCs (8.2 × 10^6) carry eCFU (3.31 × 10^-01) of *S. gordonii* (grey bubble, **c**) while circulating DCs (4.16 × 10^4) carry eCFU (4.5 × 10^-01) of *S. gordonii* (green bubble). Circulating DCs carry 42% of the eCFU carriage of *S. gordonii* (C, bi chart). The percentages (bi-chart) were calculated based on the ratio of eCFU within DCs and the total eCFU within both cells (DCs and PBMCs)

To estimate the relative capacity of different blood cell types to serve as oral consortia reservoirs, we quantified molecular signatures of the three species in ex vivo isolated circulating blood DCs and total PBMCs in PD patients. Total average eCFUs in DCs in PD patient was 4.16 × 10^4^, while the average of remaining PBMCs was 8.28 × 10^6^ (Fig. 6) (Supplemental Table 2). However, estimated *S. gordonii*, *F. nucleatum* and *P. gingivalis* counts per cell, within PanDCs were significantly higher than the counts, per cell, within PBMCs. By our calculation, circulating PanDCs carry 78% of the total estimated counts of *P. gingivalis* within PD patients’ blood (Fig. 6a). Furthermore, PanDCs carry 99% of estimated *F. nucleatum* counts within these patient’s blood (Fig. 6b). *S. gordonii* estimated counts within circulating PanDCs and PBMCs was significantly low (~4.5 × 10^−1^ CFU and 3.31 × 10^−1^ CFU), however 58% of *S. gordonii* were detected within PanDCs (Fig. 6c) (Supplemental Table 2).

## Discussion

A microbial consortium, defined as two or more microbial groups living symbiotically, was first described by Johannes Reinke in 1872.^37^ Decades of human microbiome research have since confirmed that microbes rarely live alone^38^ or function alone.^39,40^ The present study examined a three-species consortium model, reflective of the microbial pattern of tooth colonization (reviewed in^41^). *S. gordonii*, an early colonizer of the human tooth, formed a biofilm on extracted teeth. *F. nucleatum* was traditionally considered a ‘bridge-organism’ that facilitates colonization of other bacteria by coaggregation-mediated mechanisms and by promoting growth of other anaerobes;^42,43^ however, more recent studies using spectral imaging fluorescence in situ hybridization do not support a central role for *Fusobacterium* in physically connecting consortium members.^44^ *F. nucleatum* is reported to promote colorectal carcinogenesis by FadA mediated E-cadherin/beta-catenin signaling,^45^ but very little is known about its uptake by DCs. Fn has been shown to be resistant to phagocytosis by PMN, although the mechanism was not identified.^46^ Pg and Fn cocultured together or alone with mouse DCs show that Pg is the driving force for whether or not by Fn activates DCs. Although uptake was not assessed, in one study the influence of Pg on Fn was attributed to gingipains^47^ In a separate study, Fn extracts have been shown to have a suppressive effect on cell-mediated immunity and phagocyte functions, although no mechanism was not identified.^48^ *P. gingivalis*, a late colonizer, has been called a pathobiont^49^ and keystone pathogen, the latter due to its ability orchestrate inflammatory disease by remodeling a normally benign microbiota into a dysbiotic one.^29^ We showed that *P. gingivalis* alone was sparsely adherent to human teeth, but when grown as a consortium, a thick biofilm resulted, with structural similarities to those in isolated dental plaque in earlier studies.^5^ Early reports indicate that *P. gingivalis* biofilm formation is a multiple step process, involving binding of both mfa1 and fimA fimbriae and aided by *S. gordonii*.^32^ Activity of the fimA promotor is under environmental control, with decrease in fimA promotor under conditions of increased temperature, decreased hemin, and presence of saliva or serum of environmental conditions.^31^ Presence of *S gordonii* and *S. cristatus*^50^ promote or inhibit fimbriae expression respectively. While regulation of the fimA gene in *P. gingivalis* is controlled by a two-component system (FimS/FimR), regulation of mfa1 fimbriae is less well understood. Recent studies indicate that FimR is a transcriptional activator of the mfa1 gene. Unlike the regulatory mechanism of FimA by FimR, regulation of the mfa1 gene is accomplished by FimR directly binding to the promoter region of mfa1.^32^ Induction of mfa1 in *P.gingivalis* under conditions of 24 h consortium growth or when inside MoDCs suggests that these conditions may influence of FimR binding to the promoter, but this has yet to be proven. As mfa1 expression promotes survival of *P. gingivalis* within MoDCs through evasion of canonical autophagy and prevention of lysosomal fusion,^30^ this may be a factor in increased mfa1 expression in MoDCs.

Our results also suggest that there is an advantage to consortium growth for *F. nucleatum* and *S. gordonii*, but not *P. gingivalis*, at least in the short term. The interrelationships of *S. gordonii* and *P. gingivalis* in two-species consortia have been studied before, with cooperative,^51,52^ and antagonistic^53,54^ relationships reported. Many oral Streptococci, including *S. gordonii*, produce inhibitory factors such as hydrogen peroxide to inhibit growth of anaerobes.^53^ *P. gingivalis is more sensitive to peroxidases than F. nucleatum*^55^ which could explain the difference in consortium growth advantage in the presence of *S. gordonii*.

Among the most surprising and unexpected findings of our study is that *F. nucleatum* does not enter MoDCs unless in a consortium. The simplest explanation is the “Trojan horse” phenomenon, in which *F. nucleatum* is taken inside DCs by *P.gingivalis*. *P.gingivalis* readily invades DCs via its glycoprotein mfa1^34^ by targeting the C-type lectin DC-SIGN.^56^ Since we show that DC-SIGN expression is upregulated optimally by the three-species consortium, this could further account for increased uptake, although this will have to be tested experimentally. A recent review describes the concept of a “mobile microbiome,” involving the systemic spread of oral commensals and pathogens through the bloodstream to distant body sites, where they cause extra-oral infections and inflammation.^57^ These sites include human atheromatous plaques, amniotic fluid, cord blood, synovial fluid of rheumatoid arthritis, inflammatory bowel disease, colorectal cancer and respiratory tract infections, to name but a few. Of particular importance in this regard are *Streptococcus spp, Fusobacterium nucleatum* and *Porphyromonas gingivalis*. Most studies use non-cultivation-based techniques such as bacterial DNA or most recently, 16s rRNA to identify these species. The present study used 16s rRNA, but is unique in that we quantitated, by CFU-Ct regression analysis, the level of *P. gingivalis, F. nucleatum and S. gordonii* within blood PBMCs and panDCs, the latter consisting of plasmacytoid and myeloid DC subsets.

To estimate the relative carriage rate of oral consortia in panDCs or PBMCs, we calculated total counts of the three species in relation to total numbers of DCs and PBMCs in PD patients. The resultant numbers suggest that panDCs carry more bacteria per cell on average than PBMCs. By our calculation, circulating panDCs carry the majority of *P.gingivalis* and *F.nucleatum* within PD patients’ blood. These data may be reflective of the apparent higher carriage capability of DCs over PBMCs due to the diversity of C-type lectins (CTLs) expressed by immature DCs in the bloodstream. Immature DCs, by virtue of these CTLs, are specialized in the recognition and capture of pathogens, and in the polarization of the resultant effector T cell response (reviewed in^58^). Interestingly, our results also suggest that the consortium inhibits the DC maturation process that normally would downregulate C-type lectins such as DC-SIGN and convert DCs from antigen capture- to antigen-presenting cells, thus further confounding initiation of an adaptive immune response.

In conclusion we have identified a three-species oral consortium that, through polymicrobial synergy, drives microbial growth, invasion and persistence in dendritic cells (DC) and that appears to have negative consequences for the human host. It is important to reiterate in this context that the oral biofilm contains around 700 distinct species that may have other synergistic effects. Moreover, in vivo animal testing is required to confirm our in vitro and ex vivo human findings.

## Methods

### Bacterial Strains and Consortium model

*Porphyromonas gingivalis*: 381 (Pg) and *Fusobacterium nucleatum:* ATCC 49256^59^ were cultured anaerobically (10% H2, 10% CO2, and 80% N2) in Difco^TM^ Anaerobe Broth MIC (BD Biosciences San Jose, CA) in a Forma Scientific anaerobic system glove box model 1025/1029 at 37 °C. Growth curves were established for each microbe 1–4 days to determine the log (exponential phase). *Streptococcus gordonii DL1*^60^ growth curve was established in aerobic and anaerobic condition as consortium was designed to be in anaerobic condition. Hence, all experiments were carried out for each strain in anaerobic condition using Difco^TM^ Anaerobe Broth MIC (BD Biosciences San Jose, CA) in anaerobic system glove chamber. This allow unified conditions to compare each bacteria growth and gene expression as an individually or within the polymicrobial model. Growth of the three strains together was confirmed on sterile root surfaces of extracted human teeth. Initial layer of *Streptococcus gordonii* incubated at sterile human root for 2 h and then *Porphyromonas gingivalis* and *Fusobacterium nucleatum* were added for 12 h at ratio of 1 aerobes: 10 anaerobes. Samples were prepared for scanning electron microscopy (SEM) as discussed below. Complete consortium consists of the three strains, at a 1:10 aerobes to anaerobes ratio and incomplete consortium consists of two of these strains maintaining the same ratio. Mature consortium where the three strains grown together for 12 h while immature consortium was established by mixing the three strains with no time for maturation (refered to as 0 h immature consortium).

### Bacterial quantification using expression of 16s rRNA, eCFU, and qrt-PCR

eCFU were carried using expression of 16s rRNA of *P. gingivalis*, *F. Nucleatum* and *S. gordonii*. Individual TaqMan® gene expression primers were designed to target mRNA 16s rRNA of *P. gingivalis* (GenBank: AB035455.1), *F. nucleatum* (GenBank: AJ810280.1) and *S. gordonii* (GenBank: D38483.1). Forward, reverse and probe for primers were designed based on these gene sequences using online tool (Thermofisher). Each primer was verified for specificity and sensitivity by running qrt-PCR of RNA isolated from each bacterial strain with different dilutions (Supplementary fig. 1). In addition, the primer specificities were tested against RNA isolated from un-infected human MoDCs and Pan DCs (negative controls) and against infected cells with each strain (positive controls). Standard curves were generated for cycle threshold (Ct) versus known CFU values and regression analysis were carried out for each serial dilution to estimate CFU based on 16s rRNA expressions (Supplementary fig. 1). CFU for each strain were quantified based on the raw Ct values (Supplementary 1). The equations of calculating CFU for *S. gordonii were* y = 6E + 08e^−0.739×^ with *R*^2^ = 0.9753, for *F. nucleatum* y = 2E + 11e^−1.037×^ with *R*^2^ = 0.9993 and for *P. gingivalis* y = 6E + 10e^−0.867×^ with *R*^2^ = 0.8117 (Supplementary 1). For MoDCs invasion model and for quantification of bacteria within human cells each sample eCFU was normalized to the total cell count.

For qrt-PCR reaction, one-step qrt-PCR was performed using Express qPCR SuperMix (Thermofisher, Cat. no. A10312). 5 µl of the RNA sample, 25 µl PCR master mix (2×) and 2.5 µl TaqMan® gene expression assays were used per reaction. All PCRs were performed in triplicate and were carried out on a real-time PCR, StepOne® (Applied Biosystems).

Gene expression of minor (*mfa-1*) and major (*fimA*) fimbriae were quantified using 16s rRNA as housekeeping. For calculations and statistical analysis, fold changes were calculated using (2^−ΔΔCT^) method in the experimental samples.^61^ Statistical analysis for gene expression was performed using the one sample t-test, which estimates the calculated difference (in fold regulation) between experimental and control samples. A *p-*value of <0.05 is the cut-off for significant differences.

### Monocyte-derived DCs (MoDCs)

Human monocytes were isolated from mononuclear fractions of peripheral human blood by monocyte enrichment with (RossetteSep, Cat. no. 15028) for 20 min, then monocyte separation carried out by density Ficoll (GE Healthcare, Cat. no. 17-1440-03). Cells were cultured in the presence of GM-CSF (1000 unit/ml, Gemini Bio-Product, Cat. no. 300-124P) and IL-4 (1000 unit/ml, Gemini Bio-Product, Cat. no. 300-154P) at a concentration (3–4 × 105 cells/ml) for 5-6 days. Flow cytometry analyses were carried out to verify the immature DC phenotype (CD1a+, CD83−, CD14−, DC-SIGN+). Cell surface markers of DCs were evaluated by four-color immunofluorescence staining with the following antibodies: CD1a-PE (Miltenyi, Cat. no. 120-000-889), DC-SIGN-FITC (Miltenyi, Cat. no. 130-092-873), CD14-PerCP (Miltenyi, Cat. no. 130-094-969) and CD83-APC (Miltenyi, Cat. no. 130-094-186).

### MoDCs invasion model (survival and uptake of pathogen in human MoDCs)

Bacterial suspensions were washed five times in PBS and re-suspended for spectrophotometer reading at OD 660 nm of 0.11, which previously determined to be equal to 5 × 10^7^ CFU. MoDCs were pulsed with single species or consortia at 10 MOI and incubated with the MoDCs for 2, 6, 12, 24, and 48 h and each experimental condition was performed in triplicate. MoDCs were infected with mature and immature as well as complete and incomplete consortia. After MoDCs infection with the single species or consortia for 2, 6, 12, 24, and 48 h, cells were re-suspended in sterile water on ice for 20 min to lyse the cells. RNA isolation was carried out for control (uninfected cells) and experimental (infected cells) samples and eCFUs were carried out as described above.

### Time-lapse microscopy

The interaction of MoDCs with mature and immature consortia was carried out using onstage incubator microscopy (Evos FL Auto Imaging System, Thermofisher). The three species, *P. gingivalis*, *F. nucleatum* and *S. gordonii*, were grown on the lower chamber of membrane-double chamber slide (µ-Slide membrane, Ibidi GmbH) and the MoDCs were added to the upper chamber of the slide. MoDCs were cultured at the upper chamber either: (1) immediately after culturing the bacteria on the lower chamber to test their interaction with immature consortium, or (2) after 12 h of bacterial culture to test their interaction with mature consortium. Field of interest were chosen and images (at x40 magnification) were captured for 24 h for both groups (mature and immature consortia) using autofocus property. Temperature at 37 °C, humidity, and CO2 gas at 5% for normoxic condition were adjusted for the length of experiment (24 h). After image acquisition time lapse videos (Supplemental video 1 and 2) were created using software (Celleste Image Analysis, Thermofisher).

### Peripheral blood mononuclear cell (PBMCs) and circulating DCs (DC) isolation from PD patients and healthy controls

PD was defined as PPD ≥ 5 mm in ≥10 teeth, and BOP in ≥30% of sites. Periodontally healthy controls had PPD ≤ 4 mm and BOP in ≤30% sites. Periodontal assessment was carried out by two calibrated examiners (M.S.R. and A.M.F). RNA isolated from PanDCs and PBMCs then qrt-PCR was performed to detect molecular microbial signatures in each population. In addition, quantifications of Sg, Fn and Pg were carried out in the oral biofilm of healthy and PD patients. For each subject, subgingival biofilm samples were collected from the four deepest sites (one site per quadrant) that did not exhibit suppuration as observed by prior clinical examination. Before sampling, the teeth were isolated with cotton rolls and the supragingival plaque was removed. A mini-five Gracey curette (Hu-Friedy®, Chicago, IL, USA), was gently inserted into the pocket in the most apical portion and the subgingival biofilm was collected with a single stroke. The samples were placed in polypropylene tubes with 1.5 ml of a solution containing 100 ml buffer solution (10 mM Tris-HCl, 0.1 mM EDTA, pH 7.6) and stored at −80 °C until use.

### Ethical aspects

In vitro monocyte-derived DCs (MoDCs) studies were determined by the Human Assurance Committee at Augusta University to be human subject exempt, due to the use of anonymized peripheral blood samples for monocytes. Human samples in the clinical study were collected between January 2014 and May 2015 following the Helsinki Declaration of 1975, as revised in 2013. The study protocol was reviewed and approved by the Institutional Review Board of the School of Dentistry, University of São Paulo, Brazil (658.998/CEP). All subjects signed an informed consent form.

### Scanning electron microscopy (SEM)

Samples were fixed for 30–60 min in 4% paraformaldehyde, 2% glutaraldehyde in 0.1 M sodium cacodylate (NaCac) buffer, pH 7.4, postfixed in 2% osmium tetroxide in NaCac buffer, dehydrated with a graded ethanol series (25–100%), followed by a graded alcohol hexamethyldisilazane (HMDS), and the HMDS was allowed to evaporate overnight in a fume hood. The dried discs were mounted on aluminum stubs with carbon adhesive tabs and sputter coated with gold-palladium. Discs were observed and imaged in a FEI XL30 scanning electron microscope (FEI, Hillsboro, OR) at 10 kV.

### Transmission electron microscopy (TEM)

After MoDC fixation, the procedures were carried out at the Electron Microscopy and Histology Core, Department of Cellular Biology and Anatomy, Augusta University. The cells were fixed in 2% glutaraldehyde in 0.1 M sodium cacodylate (NaCac) buffer, pH 7.4, postfixed in 2% osmium tetroxide in 0.1 M NaCac, stained en bloc with 2% uranyl acetate, dehydrated with a graded ethanol series and embedded in Epon-Araldite resin. Thin sections were cut with a diamond knife and stained with uranyl acetate and lead citrate. Cells were observed in transmission electron microscope (JEM 1230–JEOL USA Inc.) at 110 kV and imaged with a CCD camera and first light digital camera controller (Gatan Inc.).

### Western blot analysis

Whole lysates were prepared from bacterial cultures by centrifuging and washing the pellet with ice-cold PBS. Proteins were extracted by adding ice-cold Radio-immunoprecipitation assay^62^ buffer (Abcam) supplemented by protease inhibitor cocktail (Cell signaling). The lysates were centrifuged at 12,000×*g* for 10 min at 4 °C. Samples were normalized to the amount of total protein in the supernatant using Pierce™ BCA Protein Assay Kit (ThermoFisher). Protein aliquots (30 μg) were separated by size on a 10% SDS-tricine-polyacrylamide gel and transferred to polyvinylidene difluoride membrane (Novex/Invitrogen, San Diego, CA). Nonspecific binding sites were blocked by incubation in 1 × TBS-T (0.2 m Tris, 0.14 m NaCl, 0.1% Tween 20) containing 2% bovine serum albumin (BSA) for 1 h at room temperature, followed by incubation overnight at 4 °C with 1:1000 dilution of mouse monoclonal anti-mfa1 antibody (in 1 × TBS-T containing 2% BSA. After membranes were washed three times in 1 × TBS-T (10 min each at room temperature), horseradish peroxidase-conjugated rabbit anti-mouse IgG (Abcam) was added at a 1:2000 dilution and incubated for 1 h at room temperature. After three more washes with 1 × TBS-T, the immunoreactive peptide was detected by Western Lightning ECL Pro Chemiluminescent reagent (PerkinElmer, Inc) and imaged using C-DiGit Blot Scanner (LI-COR).

All blots derive from the same experiment and were processed in parallel.

## Supplementary information

Immature consortium and MoDCs

mature consortium and MoDCs

Supplementary Material.

## Data Availability

The data that support the findings of this study are available from the corresponding author on reasonable request.

## Supplementary information

**Supplementary information** accompanies the paper on the *npj Biofilms and Microbiomes* website (10.1038/s41522-019-0084-7).
