# Supplementary material for: Polymicrobial synergy within oral biofilm promotes invasion of dendritic cells and survival of consortia members
Source: NPJ Biofilms Microbiomes. 2019 Mar 18;5:11. doi: 10.1038/s41522-019-0084-7 (PMC6423025; doi:10.1038/s41522-019-0084-7)

Supplementary Data

Supplementary Table 1: *fimA* and *mfa-1* Fimbriae mRNA expression in consortium *in vitro*

			Fold Regulation		
			**0 hours**	**12 hours**	**24 hours**
fimA	Pg	Avg	1.00	1.35	1.20
		Stdv	±0.3	±0.4	±0.3
	Pg+Pg	Avg	-1.17	-1.66	-1.70
		Stdv	±0.1	±0.3	±0.2
		P value	> 0.05	> 0.05	> 0.05
	Fn	Avg	2.33	1.17	2.65
		Stdv	±0.8	±0.1	±2.6
		P value	> 0.05	> 0.05	> 0.05
	Pg+Fn	Avg	-1.13	-1.89	1.09
		Stdv	±0.2	±0.1	±0.1
		P value	> 0.05	> 0.05	> 0.05
	Pg+Fn+Sg	Avg	-1.53	-1.72	-2.06
		Stdv	±0.1	±0.1	±0.2
		P value	> 0.05	> 0.05	> 0.05
mfa-1	Pg	Avg	1.40	0.75	0.95
		Stdv	±0.3	±0.5	±0.5
	Pg+Pg	Avg	-1.03	-2.58	-1.56
		Stdv	±0.0	±0.2	±0.0
		P value	> 0.05	> 0.05	> 0.05
	Fn	Avg	2.49	1.07	1.65
		Stdv	±0.1	±0.1	±0.7
		P value	> 0.05	> 0.05	> 0.05
	Pg+Fn	Avg	2.07	***-4.73**	1.09
		Stdv	±0.1	±0.3	±0.1
		P value	> 0.05	<0.01	> 0.05
	Pg+Fn+Sg	Avg	-1.70	***10.62**	***6.12**
		Stdv	±0.4	±1.2	±0.7
		P value	> 0.05	<0.01	<0.01

Fold regulations were quantified relative to controls (Pg alone) with 16S rRNA as housekeeping gene. Two-way ANOVA was used to test the time effect on fold regulation and Bonferroni post-test to compare replicate means to Pg (*P. gingivalis*) alone group. *Significant difference compared to control (Pg culture) at p<0.05. Pg: *P. gingivalis*, Sg: *S. gordonii*, Fn: *F. nucleatum*, Avg: average, Stdv: standard deviation.

Supplementary Table 2: Comparison of consortium eCFU in circulating blood DCs and PBMCs in PD patients

Consortium within circulating blood DCs				
	PanDCs counts	Pg	Fn	Sg
Avg	4.16E+04	2.95E+02	6.02E+01	4.50E-01
Stdv	1.85E+04	3.85E+02	8.87E+02	4.50E-01

Consortium within PBMCs				
	PBMCs counts	Pg	Fn	Sg
Avg	8.28E+06	8.24E+01	7.52E-01	3.31E-01
Stdv	5.26E+06	7.66E+01	4.06E-01	3.43E-01

eCFU: estimate colony forming unit, Pg: *P. gingivalis*, Sg: *S. gordonii*, Fn: *F. nucleatum*, DCs: Dendritic cells, PBMCs: peripheral blood mononuclear cell, Avg: average, Stdv: standard deviation.

Supplementary Table 3: mRNA fimbria expression in oral biofilms and in circulating blood DCs in PD patients

		Fold Regulation			
		mfa-1 mRNA		*fimA* mRNA	
		Healthy (H)	Chronic Periodontitis (CP)	Healthy (H)	Chronic Periodontists (CP)
Oral Biofilm	Avg	0.3713	91.64	4.116	164.4
	Stdv	± 1.434	± 14.18	± 2.220	± 75.33
	95% confidence interval	-117.5 to -65.07		-318.3 to -9.733	
	P value	*< 0.0001		*0.0381	
Circulating DCs	Avg	1.6713	26.61	2.3031	-36.91
	Stdv	± 3.434	± 7.239	± 1.034	± 19.08
	95% confidence interval	-39.40 to -13.07		5.196 to 69.37	
	P value	*0.0004		0.247	

Fold regulations were quantified relative to controls (Healthy samples: H) with 16S rRNA as housekeeping gene. Unpaired t test used to compare fold regulation of healthy and chronic periodontitis groups. *Significant difference relative to control (Healthy samples: H) at p>0.05. Avg: average, Stdv: standard deviation.

Supplementary Fig 2F: uncropped

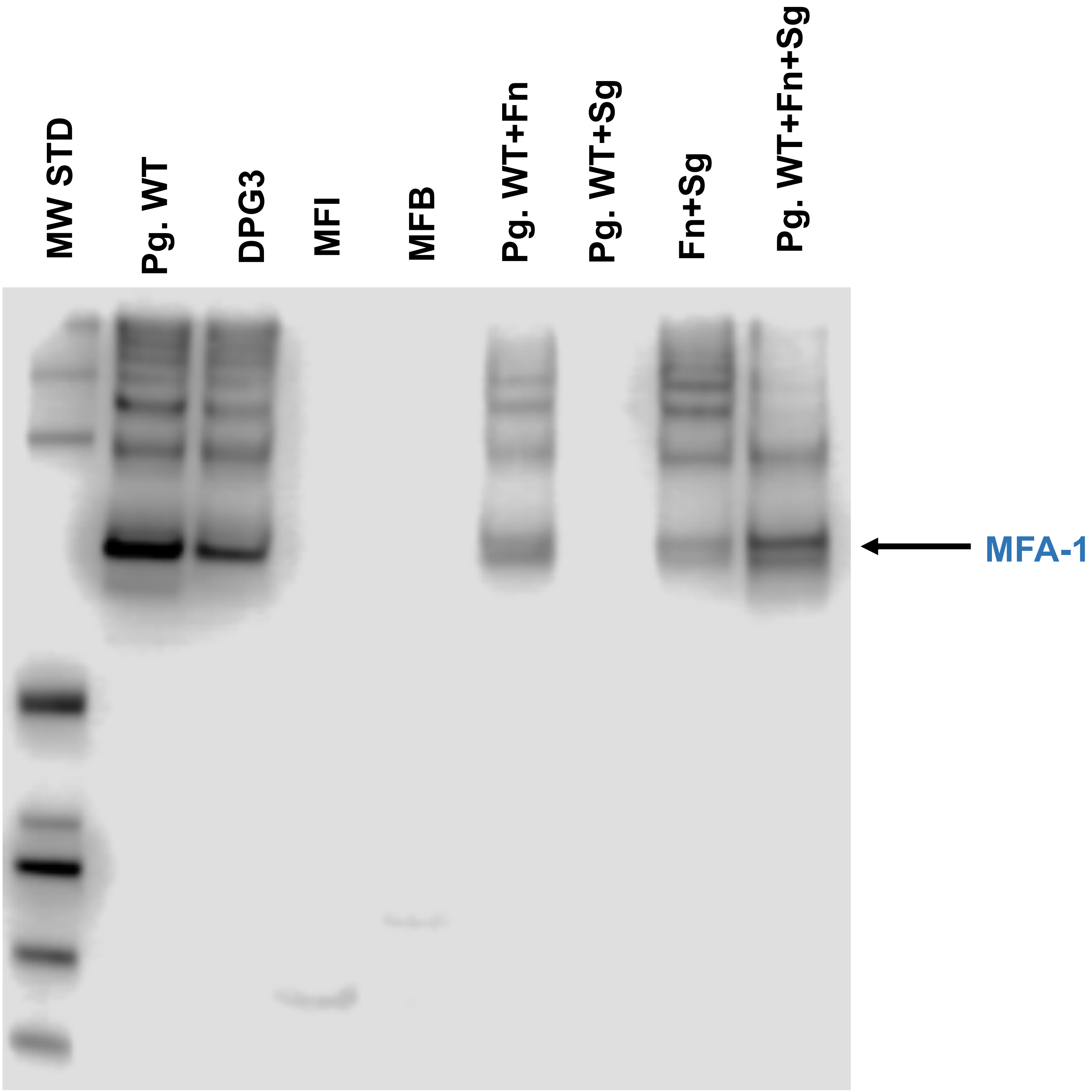

Supplement: Supplementary file 3 — Supplementary Material. [file 41522_2019_84_MOESM3_ESM.pdf]
